# Bioinformatics analysis to explore biomarkers and mechanisms of action associated with endoplasmic reticulum stress and ferroptosis in Parkinson’s disease

**DOI:** 10.1371/journal.pone.0328682

**Published:** 2025-08-08

**Authors:** Hao Wang, Lijuan Feng, Limeng He, Nan Liu, Yan Wan, Wei Zhang

**Affiliations:** 1 Department of Nuclear Medicine, Sichuan Provincial People’s Hospital, University of Electronic Science and Technology of China, Chengdu, P.R. China; 2 Department of PET/CT, Jiujiang City Key Laboratory of Cell Therapy, JiuJiang NO.1 People’s Hospital, Jiujiang, P.R. China; Indiana University School of Medicine, UNITED STATES OF AMERICA

## Abstract

**Objective:**

It has been demonstrated that Parkinson’s disease (PD) is closely associated with endoplasmic reticulum stress (ERS) and ferroptosis. However, the specific mechanisms underlying these associations remain unclear. Consequently, this study investigated the mechanisms connecting these factors and explored potential biomarkers for PD.

**Methods:**

Data for PD and ERS, as well as information on ferroptosis, were sourced from public databases and relevant literature. Candidate genes were identified through differential expression analysis and weighted gene co-expression network analysis. Further investigations included functional enrichment analysis, the construction of a protein-protein interaction (PPI) network, and the examination of related genes. Subsequently, biomarkers were validated using the least absolute shrinkage and selection operator regression algorithm. Additionally, correlations among biomarkers, gene set enrichment analysis, chromosomal and subcellular localization, immune cell infiltration, regulatory mechanisms, and drug predictions were conducted.

**Results:**

Initially, seven candidate genes were identified, predominantly associated with type II diabetes mellitus. Furthermore, five interacting associations within the PPI network and twenty related genes were identified, primarily engaged in the physical interactions pathway. Subsequently, three biomarkers were screened: N-myc downstream-regulated gene 1 (NDRG1), dihydrolipoamide dehydrogenase (DLD), and cold-inducible RNA-binding protein (CIRBP). A detailed analysis revealed a positive correlation between CIRBP and DLD, while NDRG1 exhibited a negative correlation with DLD; all three biomarkers were chiefly enriched in the oxidative phosphorylation pathway and PD. NDRG1 is located on chromosome 8, DLD on chromosome 7, and CIRBP on chromosome 19, with all three primarily localized in the nucleus. A total of 31 differential immune cells were identified between the disease and control groups, with neurons representing the highest proportion and the most significant negative correlation observed between DLD and pro B-cells. The interactions involving NORAD-hsa-miR-1277-5p-DLD, NEAT1-hsa-miR-128-3p-CIRBP, and XIST-hsa-miR-3173-5p-NDRG1 were found to be pivotal. Additionally, these biomarkers were regulated by 15 common transcription factors. Finally, nicotinamide adenine dinucleotide, pyruvic acid, nitric oxide, and phosphates were predicted as potential co-targeted therapeutic agents.

**Conclusions:**

NDRG1, DLD, and CIRBP were identified as biomarkers for PD, thereby opening new avenues for elucidating disease mechanisms, facilitating early diagnosis, and identifying potential therapeutic targets.

## 1. Introduction

Parkinson’s disease (PD) is a prevalent neurodegenerative disorder [1]. The global incidence of PD has risen significantly, with projections indicating that the number of individuals affected will increase substantially to approximately 8.7 to 9.3 million by 2030 [2,3]. As the disease progresses, it imposes a considerable burden on patients, their families, and the healthcare system. The pathogenesis of PD involves a complex interplay of factors, with the degeneration and loss of dopaminergic neurons (DN) in the substantia nigra representing the primary pathological changes [4]. Common symptoms in PD patients include resting tremor, bradykinesia, and rigidity [5]. The overall progression of PD is gradual, typically unfolding over months to years, and while the course of the disease cannot be altered, early intervention may slow its progression. Notably, most patients do not exhibit typical symptoms until the disease has advanced to a moderate or severe stage, at which point 70%–80% of the DN are already lost [6]. Despite recent advancements in the treatment of PD, significant limitations persist. While pharmacotherapy can partially alleviate symptoms, long-term use often results in reduced efficacy and adverse effects. For instance, levodopa, a mainstay treatment for PD, may lead to complications such as motor fluctuations and dyskinesia over time [7]. Additionally, PD exhibits substantial inter-individual variability in disease progression and symptom manifestation, necessitating personalized treatment plans. However, current therapeutic strategies still fall short in adequately addressing individualized needs, thereby failing to meet the requirements of all patients [8]. Therefore, a comprehensive understanding of the molecular mechanisms underlying PD development, alongside the identification and development of new diagnostic molecular markers and effective therapeutic targets, is crucial for the early diagnosis and treatment of PD patients.

The endoplasmic reticulum is essential for protein biosynthesis, folding, and assembly. When the protein load surpasses the endoplasmic reticulum’s capacity to properly fold or degrade proteins, it leads to the accumulation of unfolded and misfolded proteins, a phenomenon known as endoplasmic reticulum stress (ERS) [9]. The consequences of ERS include increased production of reactive oxygen species, altered calcium efflux, and pro-inflammatory signaling in glial cells. These pathological pathways are linked to ferroptosis, which plays a significant role in neurodegeneration [10,11]. Pharmacological targeting of these pathways may help alleviate or slow the progression of neurodegeneration. In summary, emerging evidence has highlighted a close association between PD and both ERS and ferroptosis. However, the precise mechanisms underlying these relationships remain to be fully elucidated.

Therefore, utilizing PD-related transcriptome data from public databases, this study identifies biomarkers associated with ERS and ferroptosis in PD through bioinformatics analyses, including differential expression analysis, weighted gene co-expression network analysis (WGCNA), protein-protein interaction (PPI) network construction, and machine learning. This research elucidates the molecular mechanisms that drive PD progression, thereby establishing a new theoretical foundation for future PD treatments.

## 2. Materials and methods

### 2.1. Data sources

The dataset GSE8397 was available for download from the Gene Expression Omnibus (GEO) database (https://www.ncbi.nlm.nih.gov/geo/). The sequencing platform was GPL96 based on the [HG-U133A] Affymetrix Human Genome U133A Array. The dataset contained 24 medial substantia nigra tissue samples and 15 outer tissue samples as a training set. Similarly, the dataset GSE7621 was acquired from this database. The sequencing platform was GPL570 based on the [HG-U133_Plus_2] Affymetrix Human Genome U133 Plus 2.0 Array. The dataset contained 16 nigra tissue samples about PD and 9 normal tissue samples as an independent test set. Then, 1,406 ERS-related genes [12] (S1 Table) and 431 ferroptosis-related genes [13] (S2 Table) were extracted from the literature, respectively. And a schematic overview of the study design is provided in S1 Fig.

### 2.2. Identification of candidate genes

Differentially expressed genes (DEGs) in the disease and control groups were screened in the training set according to the difference threshold (*P* < 0.05 and |log2-fold change (FC)| > 0.5) using the limma package (v 3.54.1) [14]. Subsequently, the top 10 up- and down-regulated DEGs were presented as volcano plots and heatmaps by the ggplot2 package (v 3.3.6) [15] and Complex Heatmap package (v 2.12.1) [16], respectively. In addition, WGCNA was executed on the training set using the WGCNA package (v 1.70.3) [17]. Specifically, for the accuracy of the analysis, the good Samples Genes function was first used to remove genes with a median absolute deviation in the bottom 50%, followed by the hclust function to cluster disease samples and determine the presence of outlier samples. Next, an optimal soft threshold power was determined based on the scale-free R^2^ = 0.85 and mean connectivity close to 0, which was used to construct the co-expression matrix. On the basis of the criteria of the hybrid dynamic tree cutting algorithm, the genes were categorized into different gene modules. Then, the correlation of the gene modules with the disease and control groups, respectively, was assessed by Pearson correlation, and modules with the largest positive and negative correlation coefficients were selected as key modules. Lastly, genes from these key modules were consolidated to generate the final set of key module genes. In order to obtain ERS- and ferroptosis-related differential genes, intersections were taken for DEGs, key module genes, ERS-related genes, and ferroptosis-related genes, and the obtained genes were noted as candidate genes, which were visualized by the VennDiagram package (v 1.7.1) [18].

### 2.3. Functional enrichment and PPI network analysis of candidate genes

For probing biological functions of the candidate genes, gene ontology (GO) and Kyoto Encyclopedia of Genes and Genomes (KEGG) enrichment analyses were performed, and these results were visualized by the GO plot package (v 1.0.2) [19] and the clusterProfiler package (v 4.2.2) [20]. Subsequently, the candidate genes were imported to the STRING database (http://www/string-db.org/) for constructing the PPI network (confidence score ≥ 0.4), and the network was visualized by Cytoscape software (v 3.9.0) (http://www.cytoscape.org/). Finally, GeneMANIA (http://www.genemania.org/) was applied to predict the genes related to candidate genes and pathways that were jointly involved.

### 2.4. Identification of biomarkers

In the training dataset, the least absolute shrinkage and selection operator (LASSO) analysis was performed using the “glmnet” package (v 4.1.8) [21]. Five-fold cross-validation was carried out to optimize the model performance, and Lambda.min was selected as the optimal model. Finally, feature genes were obtained from the candidate genes as potential biomarkers. Further, based on the training set, receiver operating characteristic (ROC) curves of the candidate biomarkers were plotted using the pROC package (v 1.18.5) [22] to determine the accuracy of the LASSO regression algorithm. If the area under the curve (AUC) > 0.7, it was considered high accuracy of the model. Also, an independent test set was performed to verify the model’s prediction. Lastly, differences in candidate biomarkers’ expression in the disease and control groups were compared by Wilcoxon in the training and test sets, respectively. The results were presented in the form of box plots using the ggplot2 package (v 3.3.6) [15]. And ones with remarkable differences and consistent expression trends were selected as biomarkers.

### 2.5. Correlation and functional similarity analysis and gene set enrichment analysis (GSEA) of biomarkers

In the training set, the correlation between the different biomarkers was assessed by Spearman correlation analysis with |cor| > 0.3 and *P* < 0.05. Further, scores of functional similarity between biomarkers were calculated using the GOSemSim package (v 2.24.0) [23], with a score greater than 0.5 indicating high functional similarity. At last, to realize the biological pathways involved in the biomarkers, the correlation coefficients between each biomarker and other genes were calculated by Spearman correlation analysis with |cor| > 0.3 and *P *< 0.05. And arranged in descending order to obtain a list of related genes, and subsequently, the msigdbr package (v 7.5.1) [24] was applied to fetch the background gene set (c2.cp.v7.2. symbols.gmt). The GSEA package (v 0.0.5) (https://cran.r-project.org/web/packages/GseaVis/index.html) was then employed to analyze the list of related genes in the background gene set for GSEA enrichment. Pathways with *P* < 0.05, |normalized enrichment scores (NES)| > 1, and false discovery rate (FDR) < 0.25 were defined as significantly enriched using the clusterProfiler package (v 4.2.2) [20]. Among them, the enrichment score (ES) > 0 showed a significant positive correlation, and ES < 0 showed a significant negative correlation.

### 2.6. Chromosomal and subcellular localization and immune cell infiltration analysis

It is important to understand the biomarkers localization on the chromosomes; therefore, the biomarkers localization on the chromosomes were mapped using the Circos package (v 1.2.2) [25] by downloading the gene location information from the Ensembl database (https://asia.ensembl.org/index.html). Furthermore, the amino acid sequences of the biomarker-encoded proteins were obtained from the Protein database (https://ncbi.nlm.nih.gov/protein/), and subcellular localization of the biomarker-encoded proteins were predicted using the online Uniprot database (https://www.uniprot.org/). To deeply explore the immune cell infiltration in PD, the proportion of different immune cells for each sample in the disease and control groups was assessed using the xCell package (v 1.1.0) [26] based on the training set and was demonstrated by plotting a stacked graph. Next, the Wilcoxon test was employed to determine whether immune cells obtained above were remarkably different between groups (*P* < 0.05) and was presented by plotting box plots using the ggplot package (v 3.3.6) [15]. Lastly, the correlations between biomarkers and differential immune cells were calculated using Spearman correlation analysis with |cor| > 0.3, *P* < 0.05, and presented by the pheatmap package (v 1.0.12) [16].

### 2.7. Regulatory mechanisms and drug prediction of biomarkers

Regulatory networks were of great research value and could reveal the complexity and diversity of gene expression regulation processes. Firstly, the long non-coding RNA (lncRNA)-microRNA (miRNA)-messenger RNA (mRNA) network was investigated using the multiMiR package (v 3.19) [27] for biomarkers through the diana_microt database (http://diana.imis.athena-innovation.gr/DianaTools/index.php) using the Cytoscape software (v 3.9.0) (http://www.cytoscape.org/) for visualization. Next, the ChEA3 (https://maayanlab.cloud/chea3) database was employed to find transcription factors (TFs) that regulate the biomarkers and to construct a TFs-biomarker network. In the end, the Coremine database (https://www.coremine.com) was adopted for predicting targeting therapeutic drugs for biomarkers (*P* < 0.01).

### 2.8. Statistical analysis

Bioinformatic analyses were conducted in the R software (v 4.3.1). Differences in different groups were significant at *P* < 0.05. In the box plot, ****for *P* < 0.0001, ***for *P* < 0.001, **for *P* < 0.01, *for *P* < 0.05, and ns for *P* ≥ 0.05.

## 3. Result

### 3.1. Identification of seven candidate genes

**Fig 1:**
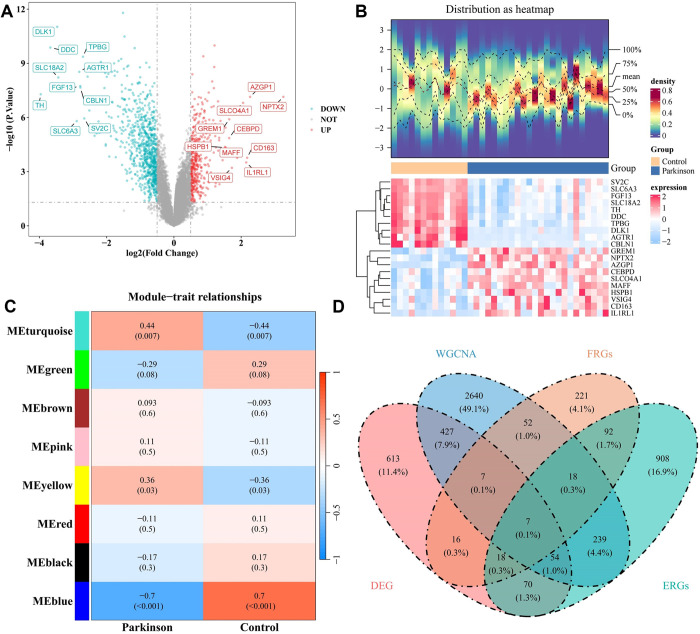
Identification and visualization of seven candidate genes. A: The volcano map displays the top 10 gene segments categorized as up-regulated and down-regulated, derived from difference analysis. B: Heatmap depict the same top 10 gene segments of up-regulated and down-regulated genes obtained through difference analysis. C: To assess the correlation between gene modules and both disease and control groups, the module with the largest negative correlation, MEblue, and the module with the largest positive correlation, MEturquoise, were selected as key modules, resulting in the identification of 3,444 key module genes. D: The Venn diagram illustrates the seven candidate genes identified through the intersection of differentially expressed genes, key module genes, endoplasmic reticulum stress-related genes, and ferroptosis-related genes.

Through the difference analysis, 1212 DEGs were totally obtained, including 424 up-regulated genes and 788 down-regulated genes, and DEGs with the top 10 up-regulated and down-regulated genes were displayed by volcano map and heatmap (Fig 1A, B). In WGCNA analysis, no outlier samples were found in the training set (S2 Fig). Subsequently, the optimal soft threshold was 3, and a total of 8 modules were acquired (S3 and S4 Fig). Among them, the absolute values of the three modules correlation were > 0.3 and *P* < 0.05, suggesting that genes within these modules could be used as differential genes of PD. Then the maximum negative correlation module of MEblue (cor = −0.7; 2126 genes) and the maximum positive correlation module of MEturquoise (cor = 0.44; 4726 genes) were regarded as the key modules, yielding 3444 key module genes (Fig 1C). Eventually, the Venn diagram displayed that there were 7 candidate genes, accounting for 0.1% of the total (Fig 1D).

### 3.2. 440 GO pathways, 152 KEGG pathways, 5 interaction associations, and 20 related genes of candidate genes

**Fig 2:**
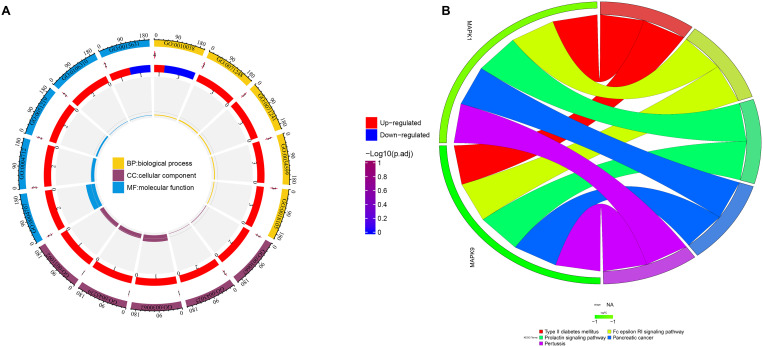
Functional enrichment analysis of candidate genes. A: The candidate genes showed enrichment in a total of 440 Gene Ontology pathways, which included 382 biological processes that are mainly activated by metal ions, 24 cellular components predominantly linked to synaptic vesicle membranes, and 34 molecular functions chiefly connected to MAP kinase activity. B: A total of 152 pathways from the Kyoto Encyclopedia of Genes and Genomes were recognized, featuring associations with type II diabetes mellitus, the Fc epsilon RI signaling pathway, and the prolactin signaling pathway.

The candidate genes were enriched in 440 GO pathways, including 382 biological processes (BP), which were mainly responsive to metal ions; 24 cellular components (CC), which were mainly enriched in synaptic vesicle membranes; and 34 that were involved in molecular functions (MF), which were mainly involved in MAP kinase activity (Fig 2A). Moreover, 152 KEGG pathways were identified, including type II diabetes mellitus, the Fc epsilon RI signaling pathway, and the prolactin signaling pathway (Fig 2B). The PPI network indicated that the 5 proteins corresponded to candidate biomarkers with a total of 5 interaction associations (S5 Fig). Ultimately, 20 related genes were predicted for the 7 candidate genes, mainly through physical interactions, co-expression, and genetic interactions in the interaction aspect and mainly oxidoreductase complex, cellular respiration, and dihydrolipoyl dehydrogenase complex in the function aspect (S6 Fig).

### 3.3. N-myc downstream-regulated gene 1 (NDRG1), dihydrolipoamide dehydrogenase (DLD), and cold-inducible RNA-binding protein (CIRBP) as biomarkers

**Fig 3:**
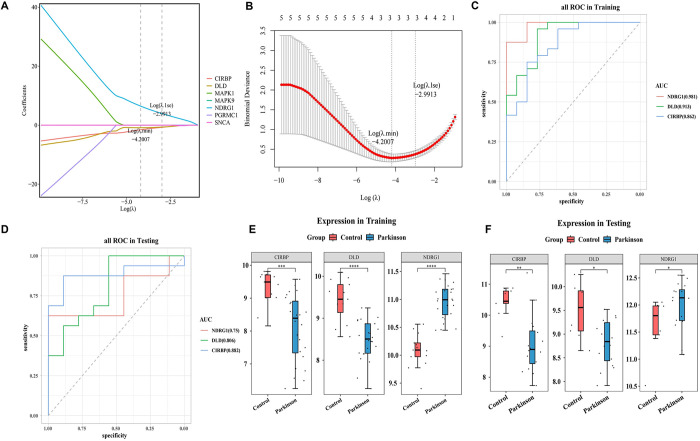
The identification of N-myc downstream-regulated gene 1 (NDRG1), dihydrolipoamide dehydrogenase (DLD), and cold-inducible RNA-binding protein (CIRBP) as biomarkers. A and B: Three genes with non-zero coefficients were selected as candidate biomarkers using the LASSO regression algorithm, specifically NDRG1, DLD, and CIRBP. C and D: The area under the curve for both the training and test sets exceeded 0.7. E and F: The expression levels of the three biomarkers were compared between the disease and control groups using the Wilcoxon test in both the training and test sets, revealing significant differences and consistent expression trends.

Three genes with non-zero coefficients were chosen as candidate biomarkers by the LASSO regression algorithm, namely NDRG1, DLD, and CIRBP (Fig 3A, B). Subsequently, AUC in the training and test sets both were > 0.7, indicating that using the LASSO regression algorithm to screen the candidate biomarkers was effective (Fig 3C, D). Eventually, due to having significant and consistent differences, NDRG1, DLD, and CIRBP were identified as biomarkers (Fig 3E, F).

### 3.4. Biomarkers having correlation and enriching in oxidative phosphorylation (OxPhos) and PD

**Fig 4:**
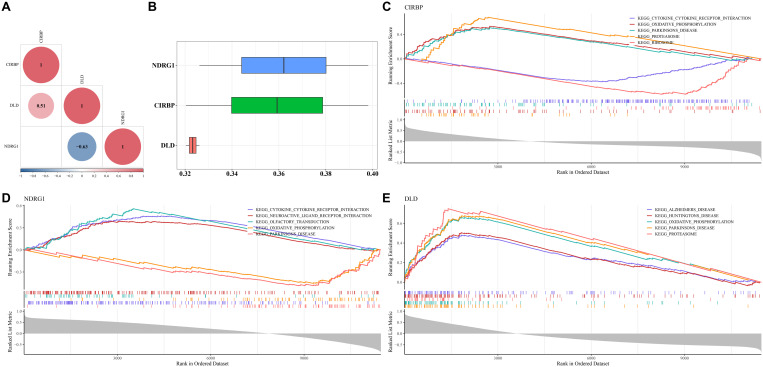
Correlation and functional similarity analysis, along with gene set enrichment analysis (GSEA), were conducted on three biomarkers. A: The correlation analysis revealed a positive correlation between CIRBP and DLD (cor = 0.51; *P* < 0.05), a negative correlation between NDRG1 and DLD (cor = −0.63; *P* < 0.05), and no correlation between CIRBP and NDRG1. B: The functional similarity among the biomarkers was found to be low (< 0.5), with NDRG1 ranging from 0.326 to 0.398, CIRBP from 0.320 to 0.398, and DLD from 0.320 to 0.326. C, D, and E: The GSEA results indicated that CIRBP, NDRG1, and DLD were all significantly enriched in oxidative phosphorylation and Parkinson’s disease.

The correlation between biomarkers indicated that there was a positive correlation between CIRBP and DLD (cor = 0.51; *P* < 0.05), a negative correlation between NDRG1 and DLD (cor = −0.63; *P* < 0.05), and no correlation between CIRBP and NDRG1 (Fig 4A). However, it could be identified that the functional similarity of biomarkers was low (< 0.5) from results of NDRG1 (0.326–0.398), CIRBP (0.320–0.398), and DLD (0.320–0.326) (Fig 4B). In the end, CIRBP, NDRG1, and DLD were all significantly enriched in OxPhos and PD (Fig 4C, D, and E).

### 3.5. Biomarkers located in the nucleus, neurons as the highest proportion of immune cells and the most correlation between DLD and pro B-cells

**Fig 5:**
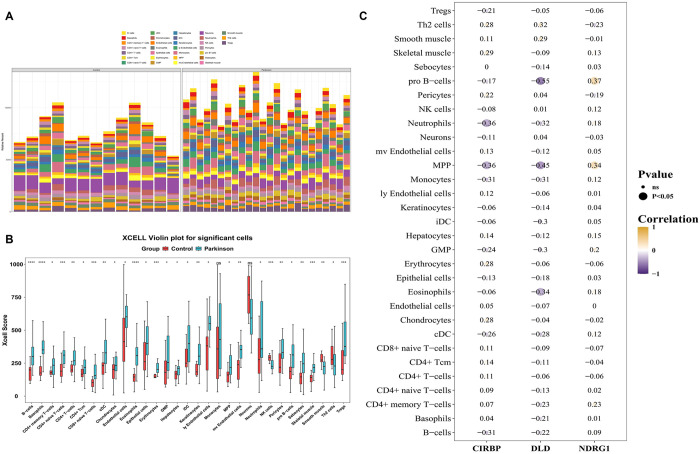
The analysis of immune cell infiltration. A and B: There were 31 differential immune cells identified between the disease and control groups, with neurons representing the highest proportion. C: CIRBP was negatively correlated with neutrophils and MPP (cor = −0.36; *P* < 0.05), while DLD exhibited a negative correlation with pro B-cells (cor = −0.55; *P* < 0.05). Conversely, NDRG1 showed a positive correlation with pro B-cells (cor = 0.37; *P* < 0.05).

Through the localization results, NDRG1 was located on chromosome 8, DLD on chromosome 7, and CIRBP on chromosome 19 (S7 Fig). Further, NDRG1, DLD, and CIRBP were all mainly located in the nucleus (S8–S10 Fig). There were 31 differential immune cells in the disease and control groups, with neurons having the highest proportion (Fig 5A, B). As shown in the heatmap, CIRBP was negatively correlated with neutrophils and MPP (cor = −0.36; *P* < 0.05), DLD with pro B-cells was negatively correlated (cor = −0.55; *P* < 0.05), and NDRG1 with pro B-cells was positively correlated (cor = 0.37; *P* < 0.05) (Fig 5C).

### 3.6. Biomarkers sharing the same target drug, including nicotinamide adenine dinucleotide (NAD), pyruvic acid, nitroxide, and phosphates

**Fig 6:**
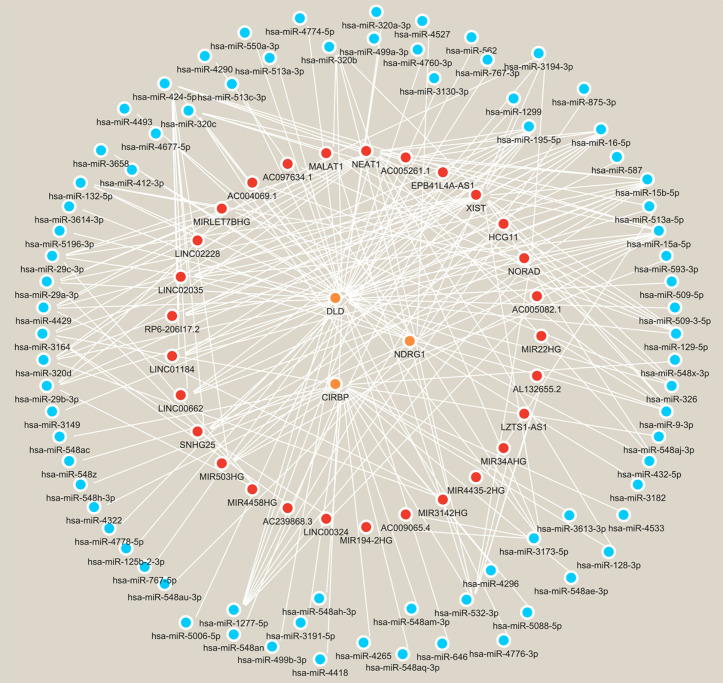
A total of 76 miRNAs were identified through the lncRNA-miRNA-mRNA network analysis. Of these, 47 miRNAs were found to regulate DLD, 21 regulated CIRBP, and 9 regulated NDRG1. Furthermore, 29 lncRNAs were identified as regulators of miRNAs. Notably, the NORAD-hsa-miR-1277-5p-DLD pathway, NEAT1-hsa-miR-128-3p-CIRBP pathway, and XIST-hsa-miR-3173-5p-NDRG1 pathway were highlighted for their significant regulatory roles.

**Fig 7:**
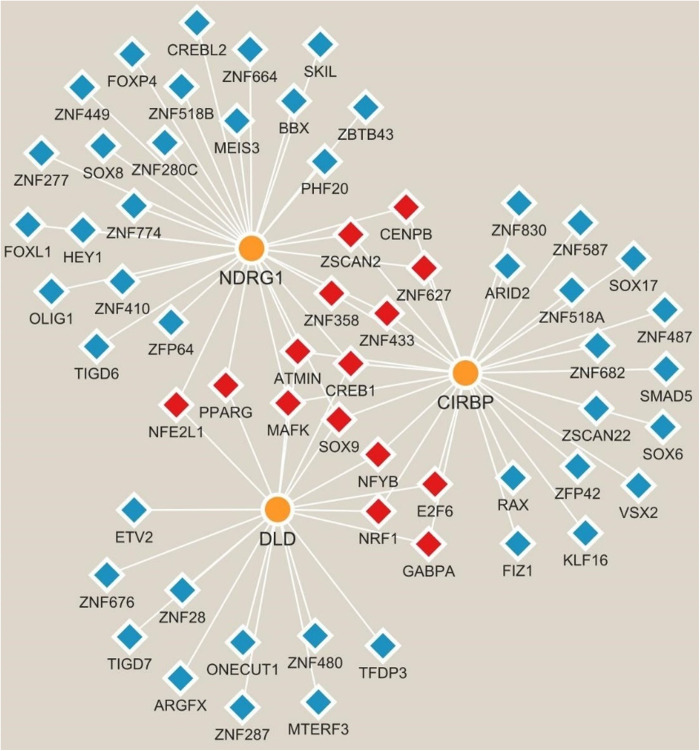
The construction of the transcription factors (TFs)-biomarker network. According to the TFs-biomarker network, 832 TFs were predicted. The top 20 TFs associated with each biomarker were displayed, resulting in a cumulative total of 60 TFs. Notably, 15 of these TFs were common across the biomarkers, including ATMIN, CENPB, CREB1, E2F6, GABPA, MAFK, NFE2L1, NFYB, NRF1, PPARG, ZNF358, ZNF433, ZNF627, SOX9, and ZSCAN2.

**Fig 8:**
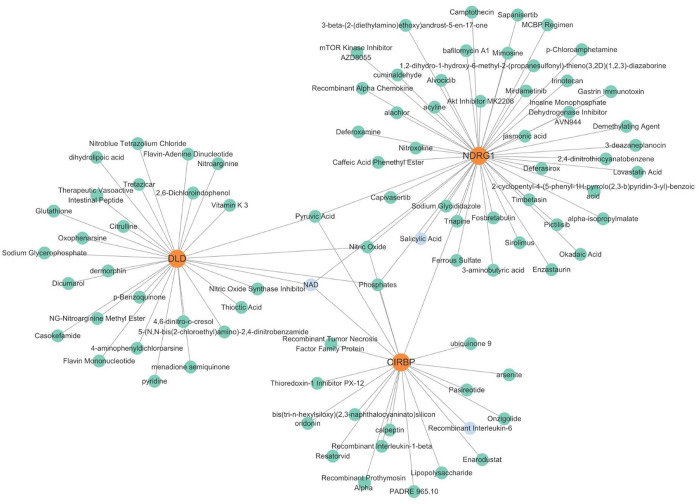
The prediction of targeted therapeutic drugs associated with various biomarkers. A total of 95 targeted therapeutic drugs were identified for these biomarkers. Specifically, 20 drugs were linked to CIRBP, with recombinant interleukin-6 receiving the highest score. Additionally, 29 drugs were associated with DLD, where NAD achieved the highest score. For NDRG1, 46 drugs were identified, with salicylic acid ranking highest. The targeted drugs shared among the three biomarkers include NAD, pyruvic acid, nitroxide, and phosphates.

There were 76 miRNAs identified by the lncRNA-miRNA-mRNA network. Among them, 47 miRNAs regulated DLD, 21 regulated CIRBP, and 9 regulated NDRG1. Additionally, 29 lncRNAs were identified to regulate miRNAs. Among them, the NORAD-hsa-miR-1277-5p-DLD pathway, NEAT1-hsa-miR-128-3p-CIRBP pathway, and XIST-hsa-miR-3173-5p-NDRG1 pathway played key regulatory roles (Fig 6). According to the TFs-biomarker network, 832 TFs were predicted. The top 20 TFs of each biomarker were displayed, totaling 60. Among them, there were 15 common TFs, including ATMIN, CENPB, CREB1, E2F6, GABPA, MAFK, NFE2L1, NFYB, NRF1, PPARG, ZNF358, ZNF433, ZNF627, SOX9, and ZSCAN2 (Fig 7). Importantly, preliminary screening identified 95 potential therapeutic agents targeting these biomarkers, though further experimental validation is required to confirm their efficacy in PD. There were 20 drugs related to CIRBP, with recombinant interleukin-6 having the highest score. There were 29 drugs related to DLD, with NAD having the highest score. There were 46 drugs related to NDRG1, with salicylic acid having the highest score. The targeted drugs shared by the three biomarkers were NAD, pyruvic acid, nitroxide, and phosphates (Fig 8).

## 4. Discussion

PD has emerged as a global public health challenge due to the rapid growth of the aging population [1]. Research on biomarkers for PD holds the potential to enable accurate diagnoses prior to the onset of widespread neuronal death [28]. Furthermore, in addition to facilitating the early diagnosis of at-risk individuals, biomarkers may also expedite drug discovery and development processes. In this study, three biomarkers associated with ERS and ferroptosis in PD—namely NDRG1, DLD, and CIRBP—were identified through various bioinformatics methods utilizing databases and existing literature. All three biomarkers exhibited significant enrichment in OxPhos pathways and were closely linked to PD. Additionally, an analysis of immune cell infiltration revealed 31 distinct immune cell types in both the disease and control groups, with neurons constituting the highest proportion. The findings of this study further suggest that NAD, pyruvic acid, nitroxide, and phosphates may serve as critical therapeutic targets for PD.

NDRG1 is a 43 kDa protein that is ubiquitously expressed in human tissues, where it plays a crucial role in regulating cell growth and differentiation [29]. NDRG1 is associated with degenerative polyneuropathy and is classified within a group of ‘late’ genes, the expression levels of which increase steadily from birth, peaking only after the completion of myelination. This observation suggests that NDRG1 has an important role in myelin maintenance. Inactivating mutations in NDRG1 can lead to severe demyelinating neuropathy [30].

DLD is a mitochondrial energy-metabolizing enzyme, and its inhibition results in elevated glucose concentrations by decreasing the conversion of pyruvic acid into the citric acid cycle [31]. Hyperglycemia increases the levels of tau protein phosphorylation, and hyperphosphorylation subsequently promotes neurodegeneration. Furthermore, DLD serves as a crucial subunit in key mitochondrial enzyme complexes such as the alpha-ketoglutarate dehydrogenase complex (KGDHC) and the pyruvate dehydrogenase complex (PDHC). Alterations in energy metabolism, including reductions in KGDHC and PDHC activity, are characteristic of many neurodegenerative diseases, including PD [32].

CIRBP is a neuroinflammatory mediator that is typically released by microglia cells in response to physiological stressors, including cerebral ischemia, alcohol exposure, and neuronal amyloid-β [33]. Research has demonstrated that CIRBP plays a role in alcohol-induced memory impairment [34]. It facilitates the generation of neurotoxic p25 via the IL-6Rα/signal transducer and activator of transcription 3/Cdk5 pathway [35]. Additionally, CIRBP promotes an increase in intracellular Ca^2+^ levels and calpain activity in neurons; both calcium dysregulation and aberrant calpain activation are mechanisms under investigation in the context of neurodegeneration [36]. Consequently, CIRBP may represent a promising new target for the treatment of neurodegenerative diseases.

GSEA enrichment analyses of biomarkers predominantly highlight OxPhos and PD. DNs are highly arborized and exhibit redundancy, necessitating more energy than other types of neurons. Normal mitochondrial function is essential for the survival of DNs. OxPhos serves as the primary pathway for energy production within mitochondria [37]. Mitochondria are critical for supplying adenosine triphosphate (ATP) to the cell through OxPhos, as well as for the synthesis of essential biomolecules [38]. Various redox reactions, catalyzed by enzymes, occur during OxPhos [39]. Inefficient OxPhos can generate reactive oxygen species, leading to mitochondrial dysfunction, which is regarded as a significant factor in the pathogenesis of neurodegenerative diseases. Many genes associated with PD are linked to OxPhos, particularly complex I [37]. The deletion of complex I results in decreased ATP formation in these neurons, and diminished ATP concentrations may lead to membrane depolarization, disruption of calcium signaling, opening of the permeability transition pore, and reduced fission rates, thereby promoting neurodegeneration. Complex I also plays a vital role in regulating the permeability transition pore. Inhibition of complex I results in increased formation of reactive oxygen species and altered fission-fusion dynamics, ultimately leading to cell death [40].

Neurons constituted the highest proportion of immune cells in both the disease and control groups. Although neurons are not considered ‘professional’ immune cells, they exhibit innate immune responses. Neurons express phagocytic and scavenger receptors that can differentiate between ‘self’ (host) and ‘nonself’ (neurotoxic proteins, pathogens, and apoptotic cells), thereby minimizing bystander damage [41]. Additionally, neurons release ‘death signals’ that trigger apoptosis in damaged neurons and inflammatory cells, effectively transforming them into ‘safe targets’ for swift clearance by glial cells that express phagocytic receptors. This process not only contributes to brain defenses but also facilitates the removal of neurotoxic proteins and apoptotic cells from the central nervous system, thereby promoting tissue repair and the rapid restoration of tissue homeostasis.

In addition, this study confirmed that the targeted drugs associated with the three biomarkers were NAD and pyruvic acid. NAD is a crucial metabolite involved in cellular bioenergetics, genome stability, mitochondrial homeostasis, adaptive stress responses, and cell survival [42]. Numerous NAD-dependent enzymes play a role in synaptic plasticity and neuronal stress resistance [43]. The pathology of PD is characterized by a dual loss of neuronal energy metabolism and an increase in oxidative stress. Pyruvic acid offers protection against neuronal damage by serving both as a metabolic energy fuel for neurons and by rapidly undergoing decarboxylation, acting as an antioxidant released by brain glial cells [44]. Studies have demonstrated the therapeutic potential of pyruvic acid in neurodegenerative lesions, with nearly complete prevention of neuronal injury observed following intraperitoneal injection of pyruvic acid one hour after reperfusion in rats subjected to transient forebrain ischemia [45]. In vitro studies indicate that cortical neurons prefer pyruvic acid over glucose as an energy substrate, with pyruvic acid reducing excitotoxic injury and preventing intra-neuronal calcium overload. The introduction of pyruvic acid into glucose-deprived neurons has been shown to restore excitatory postsynaptic potentials and mitigate neuronal damage.

Previous studies have identified several biomarkers related to PD, but these have largely been confined to ferroptosis-related genes, with their specific mechanisms of action in the disease remaining unclear [46–49]. In this study, we integrated genes related to ERS and ferroptosis to comprehensively analyze gene expression changes in PD. Furthermore, we conducted a series of analyses on the identified biomarkers (NDRG1, DLD, and CIRBP), including chromosome localization, functional similarity analysis, tissue localization analysis, GSEA functional enrichment, immune infiltration analysis, regulatory network construction, and drug network construction. These analyses have enhanced our understanding of the roles of ERS and ferroptosis in the pathogenesis of PD and further elucidated the potential mechanisms of these biomarkers in disease progression.

The study has several limitations. The relatively small sample size may limit the generalizability of our findings. Additionally, the lack of detailed racial and genetic background information in the public datasets further restricts the population applicability of our conclusions. Furthermore, reliance solely on genomic data may not fully elucidate the complex molecular mechanisms underlying neurodegenerative diseases such as PD. To address these limitations, we plan to utilize larger-scale and more diverse genetic datasets in future research. We will also integrate multi-omics approaches, including genomics, proteomics, and metabolomics, to achieve a more comprehensive understanding of disease mechanisms. Currently, we are collaborating with multiple research centers and intend to validate our findings through functional experiments and independent cohort studies. Moreover, although we have predicted potential therapeutic agents, these findings remain speculative until they are experimentally validated. Therefore, we plan to conduct in vitro studies to assess the effects of the prioritized candidate drugs on neuronal models of PD.

## 5. Conclusions

This study employed comprehensive bioinformatics approaches to identify NDRG1, DLD, and CIRBP as key biomarkers associated with both ERS and ferroptosis in PD. These findings provide crucial molecular insights into PD pathogenesis, potentially paving the way for improved early diagnosis strategies and the identification of novel therapeutic targets. Further analysis elucidated complex regulatory networks involving these biomarkers, including critical lncRNA-miRNA-mRNA interactions (e.g., NORAD-hsa-miR-1277-5p-DLD, NEAT1-hsa-miR-128-3p-CIRBP, XIST-hsa-miR-3173-5p-NDRG1) and shared transcriptional regulation by 15 common transcription factors (e.g., ATMIN, CREB1, NRF1). Importantly, drug prediction analyses identified NAD, pyruvic acid, nitroxide, and phosphates as potential co-targeted therapeutic agents acting on these biomarkers, suggesting avenues for intervention targeting the ERS-ferroptosis axis in PD. While these bioinformatics findings offer significant new perspectives, acknowledged limitations include the sample size. As bioinformatics tools and disease databases continue to evolve, enhancing data reliability, further validation through larger genetic datasets and experimental studies is essential to solidify these conclusions and translate them into clinical applications.

## Supporting information

S1 Fig A schematic overview of the study design. (PDF)

S2 Fig Cluster of disease samples by the hclust function of WGCNA. Each branch represented an individual sample, while the vertical axis indicated the height of hierarchical clustering. The upper section of the diagram displayed the sample branches, whereas the lower section illustrated the expression scores of directional genes across these samples.
(PDF)

S3 Fig Determination of an optimal soft threshold power to construct the co-expression matrix. The horizontal axes in both the left and right plots represented the weighting parameter (power value).
(PDF)

S4 Fig Categorization of gene modules on the basis of the criteria of the hybrid dynamic tree cutting algorithm. The upper section displayed a hierarchical clustering dendrogram of module genes, where genes with closer relationships exhibited higher similarity in branch height. The lower section used distinct color blocks to represent different functional modules.
(PDF)

S5 Fig Construction of the PPI network. Nodes represented genes, and connecting edges indicated interactions between genes.
(PDF)

S6 Fig Prediction of the genes related to candidate genes and pathways that were jointly involved. Nodes represented genes, connecting edges indicated interactions between genes (with different colors denoting distinct interaction types), and node colors corresponded to different functional categories.
(PDF)

S7 Fig Mapping of the location of 3 biomarkers on chromosomes. (PDF)

S8 Fig Prediction of subcellular localizations of the NDRG1-encoded proteins. (PDF)

S9 Fig Prediction of subcellular localizations of the DLD-encoded proteins. (PDF)

S10 Fig Prediction of subcellular localizations of the CIRBP-encoded proteins. (PDF)

S1 Table 1,406 ERS-related genes extracted from the literature. (CSV)

S2 Table 431 ferroptosis-related genes extracted from the literature. (CSV)
