# Supplementary figures and images for: Bioinformatics analysis to explore biomarkers and mechanisms of action associated with endoplasmic reticulum stress and ferroptosis in Parkinson’s disease
Source: PLoS One. 2025 Aug 8;20(8):e0328682. doi: 10.1371/journal.pone.0328682 (PMC12333997; doi:10.1371/journal.pone.0328682)

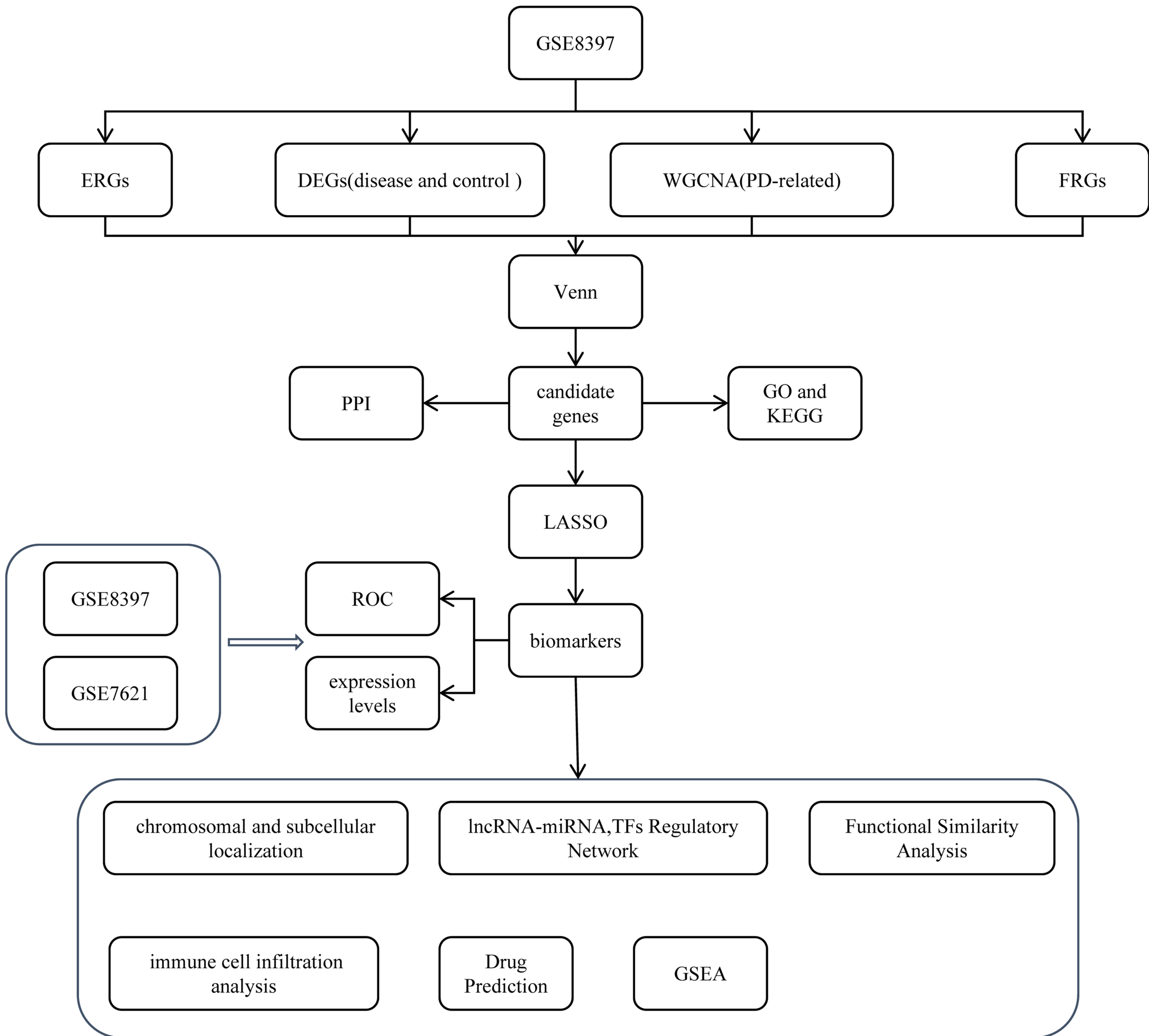

Supplement: S1 Fig — (PDF) [file pone.0328682.s001.pdf]

# Sample Clustering and trait heatmap

Height

20 40 60 80 100

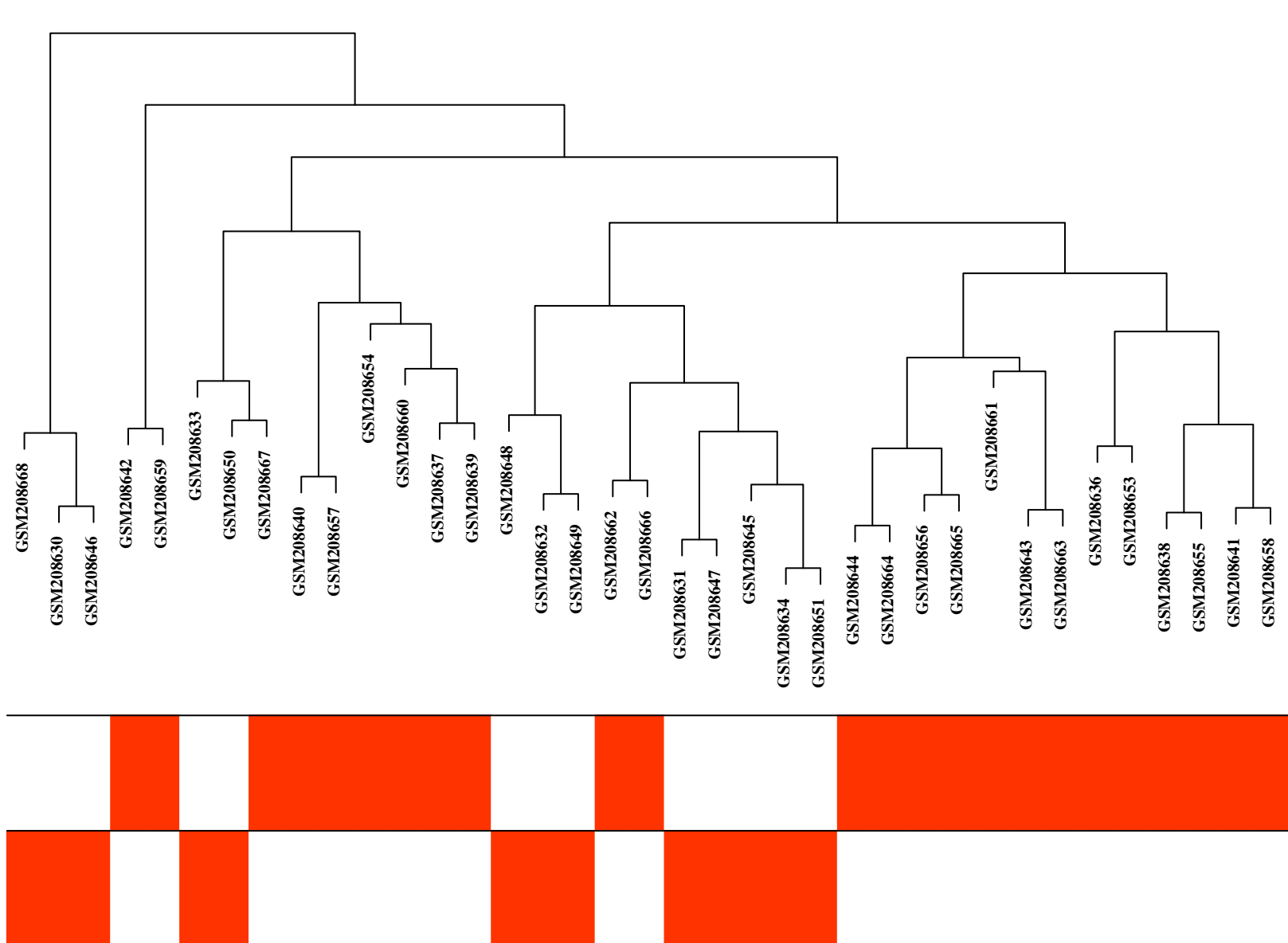

Supplement: S2 Fig — Each branch represented an individual sample, while the vertical axis indicated the height of hierarchical clustering. The upper section of the diagram displayed the sample branches, whereas the lower section illustrated the expression scores of directional genes across these samples. (PDF) [file pone.0328682.s002.pdf]

### Scale independence

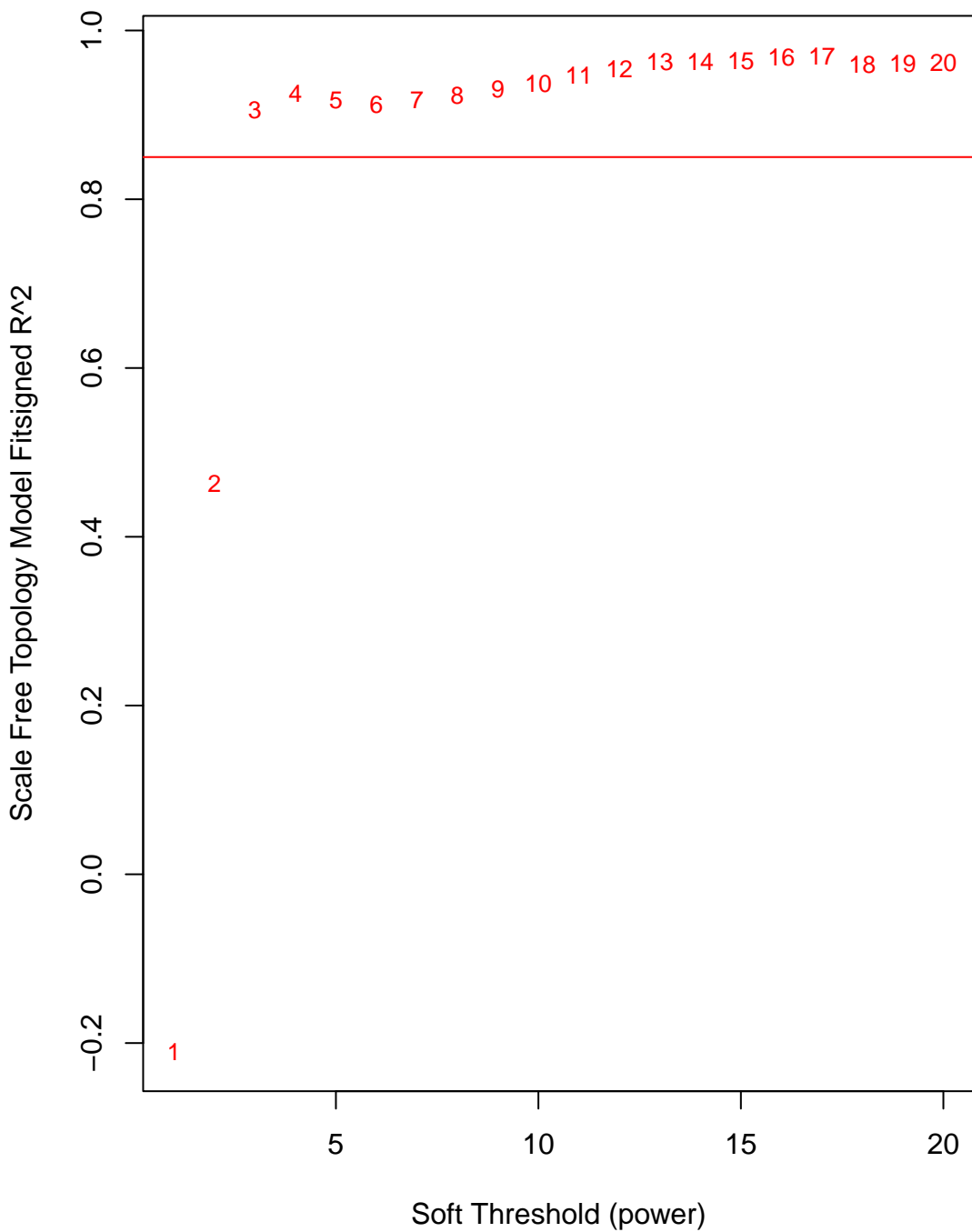

### Mean connectivity

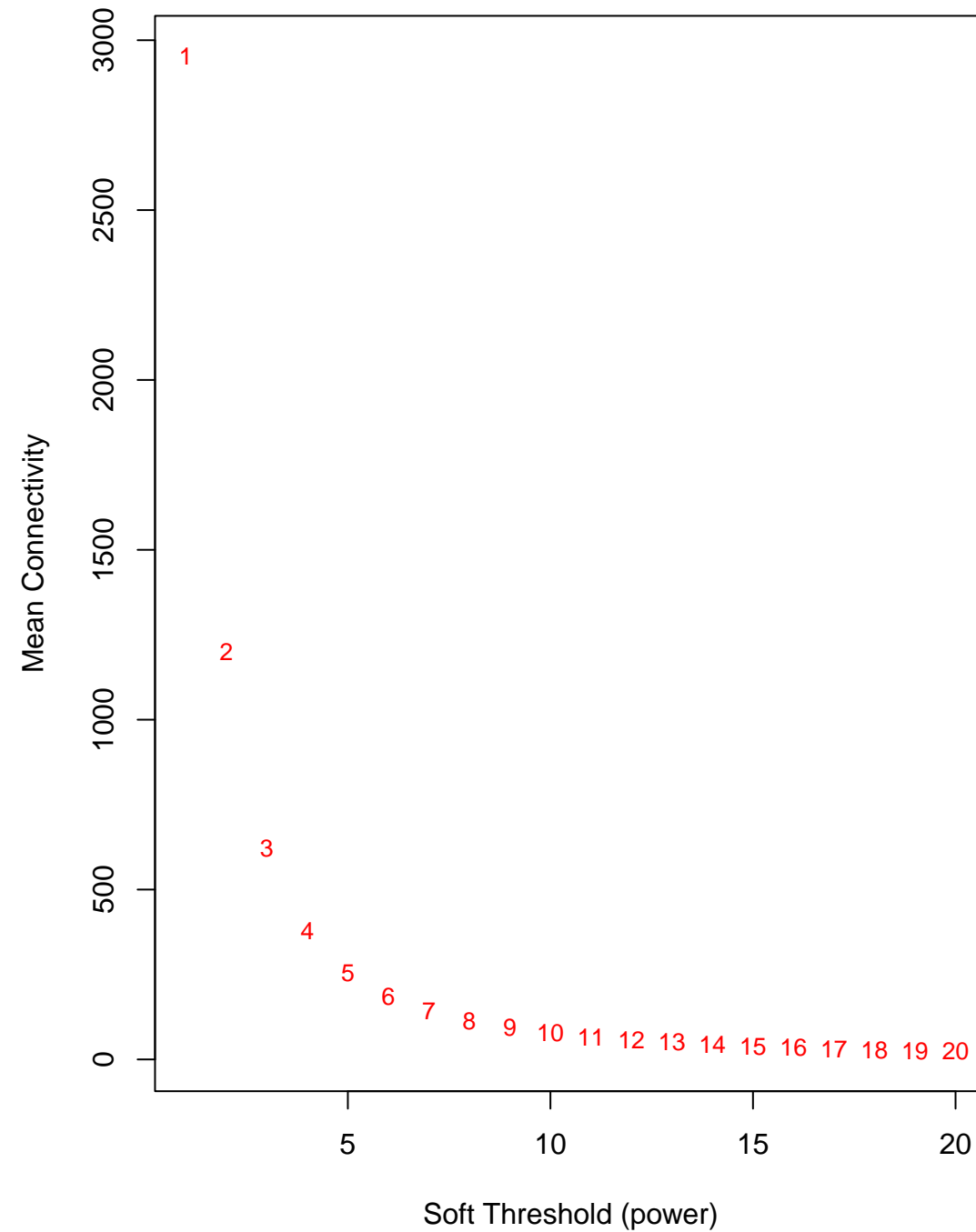

Supplement: S3 Fig — The horizontal axes in both the left and right plots represented the weighting parameter (power value). (PDF) [file pone.0328682.s003.pdf]

# Cluster Dendrogram

Height

0.8  
0.7  
0.6  
0.5  
0.4

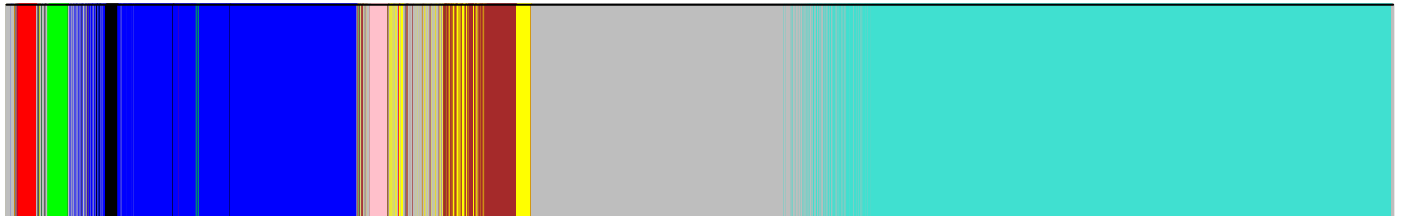

Supplement: S4 Fig — The upper section displayed a hierarchical clustering dendrogram of module genes, where genes with closer relationships exhibited higher similarity in branch height. The lower section used distinct color blocks to represent different functional modules. (PDF) [file pone.0328682.s004.pdf]

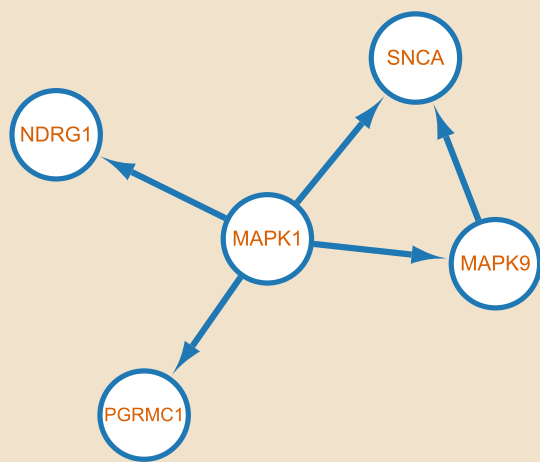

Supplement: S5 Fig — Nodes represented genes, and connecting edges indicated interactions between genes. (PDF) [file pone.0328682.s005.pdf]

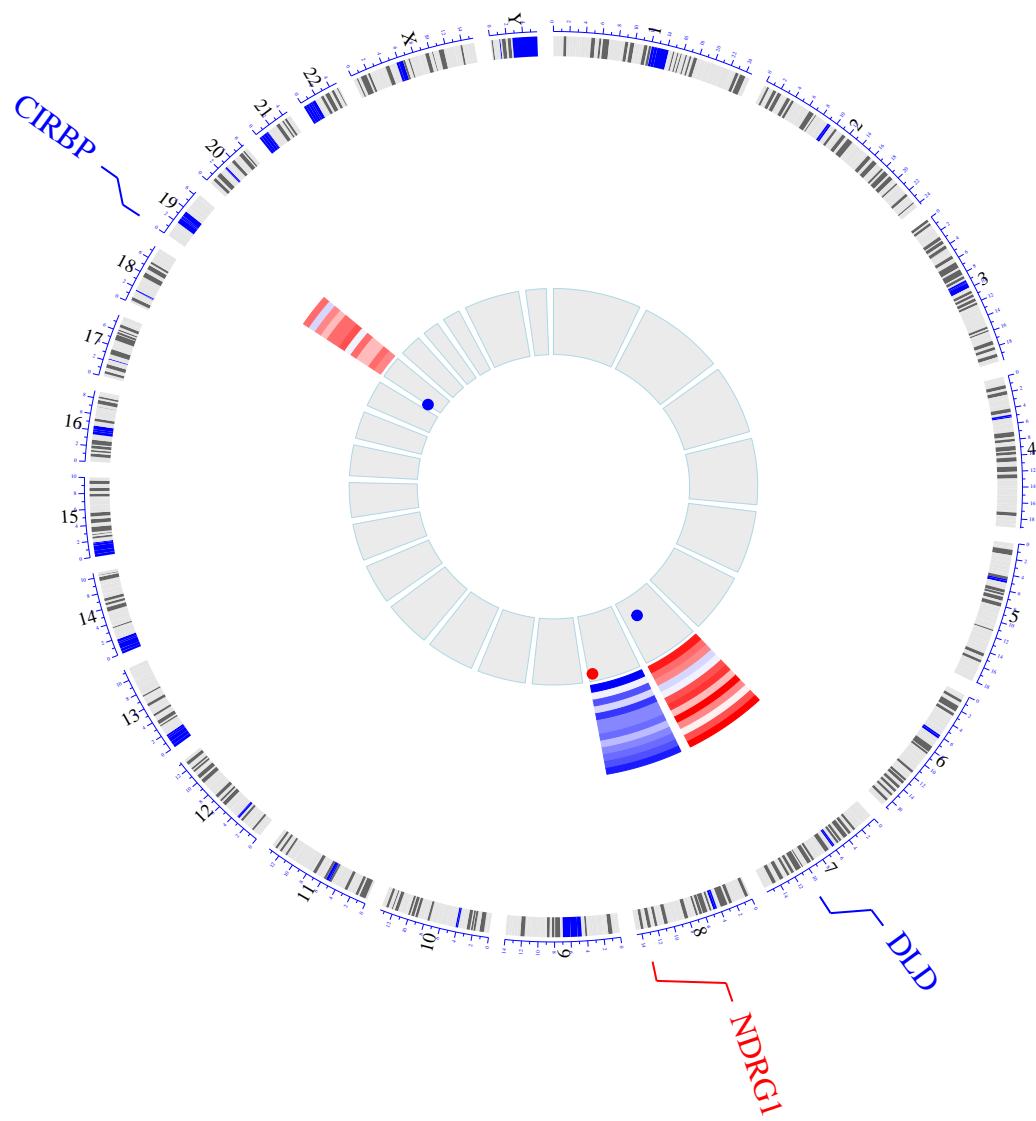

Supplement: S7 Fig — (PDF) [file pone.0328682.s007.pdf]

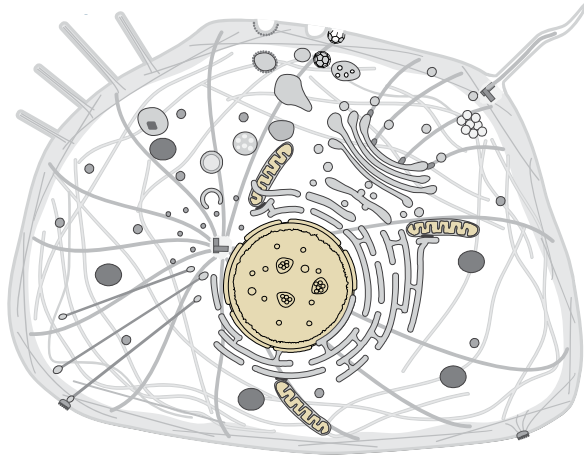

📍 **Mitochondrion matrix**

📍 **Nucleus**

Supplement: S9 Fig — (PDF) [file pone.0328682.s009.pdf]

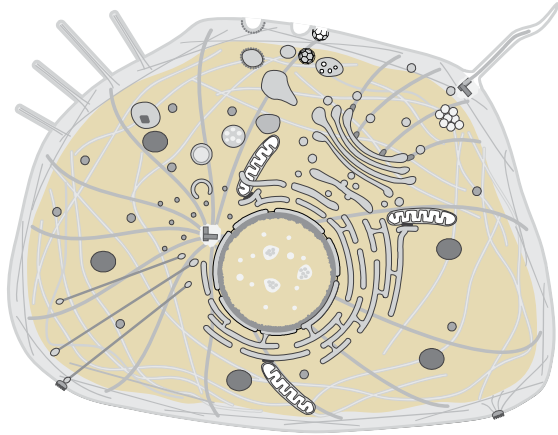

📍 **Nucleus, nucleoplasm**

📍 **Cytoplasm**

Supplement: S10 Fig — (PDF) [file pone.0328682.s010.pdf]
