# Supplementary material for: Bioinformatics analysis to explore biomarkers and mechanisms of action associated with endoplasmic reticulum stress and ferroptosis in Parkinson’s disease
Source: PLoS One. 2025 Aug 8;20(8):e0328682. doi: 10.1371/journal.pone.0328682 (PMC12333997; doi:10.1371/journal.pone.0328682)
Supplement: S8 Fig — (PDF) [file pone.0328682.s008.pdf]

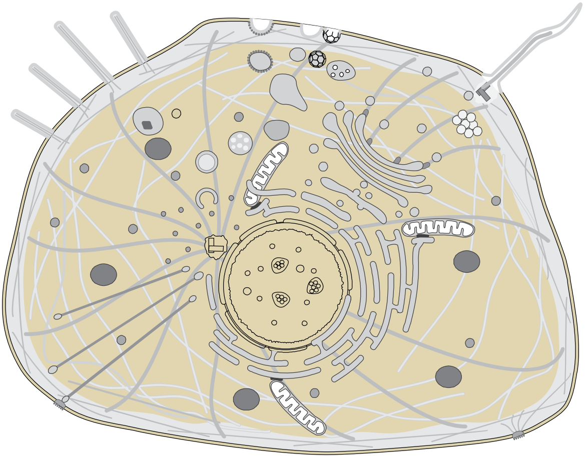

- Cytoplasm, cytosol
- Cytoplasm, cytoskeleton, microtubule organizing center, centrosome
- Nucleus
- Cell membrane
